# Effects of Apigenin on RBL-2H3, RAW264.7, and HaCaT Cells: Anti-Allergic, Anti-Inflammatory, and Skin-Protective Activities

**DOI:** 10.3390/ijms21134620

**Published:** 2020-06-29

**Authors:** Che-Hwon Park, Seon-Young Min, Hye-Won Yu, Kyungmin Kim, Suyeong Kim, Hye-Ja Lee, Ji-Hye Kim, Young-Jin Park

**Affiliations:** 1 Department of Medicinal Biosciences, Research Institute for Biomedical & Health Science, College of Biomedical and Health Science, Konkuk University, 268 Chungwon-daero, Chungju-si 27478, Korea; chehwon9798@kku.ac.kr (C.-H.P.); 124msy@kku.ac.kr (S.-Y.M.); ryu1hw@kku.ac.kr (H.-W.Y.); 2 Jeju R&D Center, AMI Cosmetics Co., Ltd., 16, Sancheondandong-gil, Jeju-si 63359, Korea; rainant@skinami.co.kr (K.K.); cjksy25@skinami.co.kr (S.K.); 3 Natural Products Laboratory, DAEBONG Life Science Co., Ltd., 213-4, Chumdan-Ro, Jeju-si 63309, Korea; hj4170@daebongls.co.kr (H.-J.L.); jh.kim2@daebongls.co.kr (J.-H.K.)

**Keywords:** anti-inflammation, anti-allergy, apigenin, flavone, RBL-2H3, RAW264.7, HaCaT

## Abstract

Apigenin (4′,5,7-trihydroxyflavone, flavonoid) is a phenolic compound that is known to reduce the risk of chronic disease owing to its low toxicity. The first study on apigenin analyzed its effect on histamine release in the 1950s. Since then, anti-mutation and antitumor properties of apigenin have been widely reported. In the present study, we evaluated the apigenin-mediated amelioration of skin disease and investigated its applicability as a functional ingredient, especially in cosmetics. The effect of apigenin on RAW264.7 (murine macrophage), RBL-2H3 (rat basophilic leukemia), and HaCaT (human immortalized keratinocyte) cells were analyzed. Apigenin (100 μM) significantly inhibited nitric oxide (NO) production, cytokine expression (interleukin (IL)-1β, IL6, cyclooxygenase (COX)-2, and inducible nitric oxide synthase [iNOS]), and phosphorylation of mitogen-activated protein kinase (MAPK) signal molecules, including extracellular signal-regulated kinase (ERK) and c-Jun N-terminal protein kinase (JNK) in RAW264.7 cells. Apigenin (30 μM) also inhibited the phosphorylation of signaling molecules (Lyn, Syk, phospholipase Cγ1, ERK, and JNK) and the expression of high-affinity IgE receptor FcεRIα and cytokines (tumor necrosis factor (TNF)-α, IL-4, IL-5, IL-6, IL-13, and COX-2) that are known to induce inflammation and allergic responses in RBL-2H3 cells. Further, apigenin (20 μM) significantly induced the expression of filaggrin, loricrin, aquaporin-3, hyaluronic acid, hyaluronic acid synthase (HAS)-1, HAS-2, and HAS-3 in HaCaT cells that are the main components of the physical barrier of the skin. Moreover, it promoted the expression of human β-defensin (HBD)-1, HBD-2, HBD-3, and cathelicidin (LL-37) in HaCaT cells. These antimicrobial peptides are known to play an important role in the skin as chemical barriers. Apigenin significantly suppressed the inflammatory and allergic responses of RAW264.7 and RBL cells, respectively, and would, therefore, serve as a potential prophylactic and therapeutic agent for immune-related diseases. Apigenin could also be used to improve the functions of the physical and chemical skin barriers and to alleviate psoriasis, acne, and atopic dermatitis.

## 1. Introduction

Studies have been directed to discover bioactive ingredients with physiological activities such as phytochemicals from various natural resources. Natural substances exert fewer side-effects than conventional synthetic materials [1], and can effectively inhibit the overproduction of reactive oxygen species (ROS) such as unstable and highly reactive free radicals, thereby preventing mutation and cytotoxicity [2]. Oxidative stress has been reported to promote diseases or aging by inducing damage to cells and tissues. Hence, increasing attention has been diverted to the development of products that reduce oxidative stress [3,4,5]. In this direction, potent bioactive substances have been discovered from plants and have been actively applied to functional foods, pharmaceuticals, and cosmetics [6,7].

Inflammation caused by infections or tissue injury is associated with various human diseases. Neutrophils produce ROS, reactive nitrogen species (RNS), and nitric oxide (NO), all of which are inflammation-inducing agents [8]. The incidence of atopic dermatitis (AD), of all the skin diseases, is gradually increasing. AD is caused by intrinsic and extrinsic factors related to the skin. Acute or chronic skin damage allows easy access of various allergens to the skin, resulting in an increase in allergic inflammatory responses [9,10,11]. Among various inflammatory mediators, interleukin (IL)-4 and IL-13 produced following the activation of type-2 helper T cells (Th2) are known to be overexpressed and induce immunoglobulin E (IgE) production in patients with allergic dermatitis and AD [12,13,14]. Cells that play the most important role in inducing allergic reactions are mast cells and basophils, which mediate allergic reactions by secreting β-hexosaminidase. IgE binds to the IgE-binding subunit of the high-affinity IgE receptor (FcεRI), a heterotetrameric receptor (one α, one β, and two γ subunits), on mast cells and basophils and promotes degranulation and cytokine secretion, leading to an allergic reaction [15,16,17]. IgE binding to the α-subunit results in the activation of the β- and γ-subunits of the FcεRI, consequently recruiting Lyn and Syk and inducing phosphorylation of protein tyrosine kinases (PTKs). Activated Syk is shown to be involved in the phosphorylation and activation of phospholipase C (PLC)-γ [15,16,17]. Mitogen-activated protein kinases (MAPKs) such as extracellular signal-regulated kinase (ERK), c-Jun N-terminal protein kinase (JNK), and p38 are also activated by FcεRI-IgE crosslinking. Phosphorylation and activation of these MAPKs mediate the expression of tumor necrosis factor-α (TNF-α) and IL-2 [18,19,20,21,22]. Macrophages play an important role in immune response and regulate various inflammatory mediators such as NO, prostaglandin (PG), and preinflammatory cytokines [23]. In mammals, there are three types of nitric oxide synthases (NOS), of which type III inducible NOS (iNOS) is expressed only in response to stimuli such as lipopolysaccharide (LPS), cytokines, and bacterial toxins in some cells [24,25]. NO is mainly produced by iNOS and promotes inflammatory responses by inducing the expression of inflammatory mediators [26]. Another inflammatory factor, cyclooxygenase (COX), is an enzyme that converts arachidonic acid into PG. There are two types of COX, COX-1, and COX-2, that exhibit different expression patterns in various cells. COX-2 is primarily expressed during the inflammatory response and induces the production of prostaglandin E2 (PGE2), an inflammatory mediator associated with pain and fever [27]. In addition, one of the cytokines, TNF-α, plays an important role in the induction of inflammatory reactions through the activation of T cells and macrophages and enhancement of other pro-inflammatory cytokines [28], leading to an inflammatory response. IL-6 is also an important inflammatory factor secreted by macrophages upon LPS exposure [29].

The skin comprises the epidermis, dermis, and subcutaneous tissue (hypodermis) and acts as a barrier to maintain moisture. It prevents the invasion of external infectious agents through the production of antimicrobial peptides [30,31]. In particular, keratinocytes constituting the epidermis produce involucrin, loricrin, and filaggrin to aggregate keratin filaments and form a cornified cell envelope [32]. In addition, hyaluronic acid (HA) synthesized by hyaluronic acid synthase (HAS) in keratinocytes and fibroblasts also functions as a moisturizing barrier in the skin [33]. Aquaporins (AQPs) are small hydrophobic integral membrane proteins that regulate the water retention rate of skin and other organs. To date, 13 types of AQPs have been identified in mammals [34], of which AQP3 is involved in the transport of water and glycerol. Human epidermal keratinocytes express AQP3 on their membranes [35]. In mice lacking AQP3 expression, the water transport capacity of the collecting duct was found to be reduced by about three times, consistent with the occurrence of polyuria and delayed wound healing owing to increased skin dryness [36,37,38,39]. Keratinocytes also produce antimicrobial peptides such as defensin (human β-defensin (HBD)-1, HBD-2, HBD-3), cathelicidin, secretory leukocyte proteinase inhibitor, dermcidin, and adrenomedullin for defense against infectious agents. Chronic skin disease in response to high bacterial infection rate has been reported to be associated with the decreased expression of these antimicrobial peptides [40,41,42,43]. The abnormalities of these skin cell components and antimicrobial peptides are also presumed to be the cause of AD along with other factors such as genetic and environmental factors and imbalance in the immune response [44,45,46].

While screening various natural resources, we found that barley sprout exhibits excellent anti-inflammatory and anti-allergic activities [47]. Barley (*Hordeum vulgare* L.) is a major crop belonging to the Poaceae (Gramineae) family. In particular, barley leaves are rich in various bioactive substances such as vitamin C, vitamin E, catechin, kaempferol, quercetin, and β-carotene. Studies have been performed to analyze the nutritional value and various physiological activities of barley, but no study has systematically evaluated the different beneficial properties of barley [48,49,50,51]. According to the flavonoid database 1.0 [52,53], barley sprout contains a relatively higher level of apigenin (4′,5,7-trihydroxyflavone, flavonoid), a type of phenolic compound, than other crops. Apigenin exerts health-promoting effects and is known to reduce the risk of chronic disease owing to its low toxicity [54,55,56]. Further, apigenin has been reported to exhibit remarkable effects against cancerous cells [55,56].

To confirm the applicability of natural resources, it is imperative to prove the effectiveness of the main ingredients contained in the resources. Therefore, this study aimed to evaluate apigenin, the main ingredient of barley sprout, for its anti-allergic effects on basophils (RBL-2H3) and anti-inflammatory effects on macrophages (RAW264.7). In addition, we investigated the effects of apigenin on human epidermal keratinocytes (HaCaT) to determine its potential as a natural substance for the prevention of AD.

## 2. Results and Discussion

### 2.1. Cytotoxicity of Apigenin in RAW264.7, RBL-2H3, and HaCaT Cells

**Figure 1:**
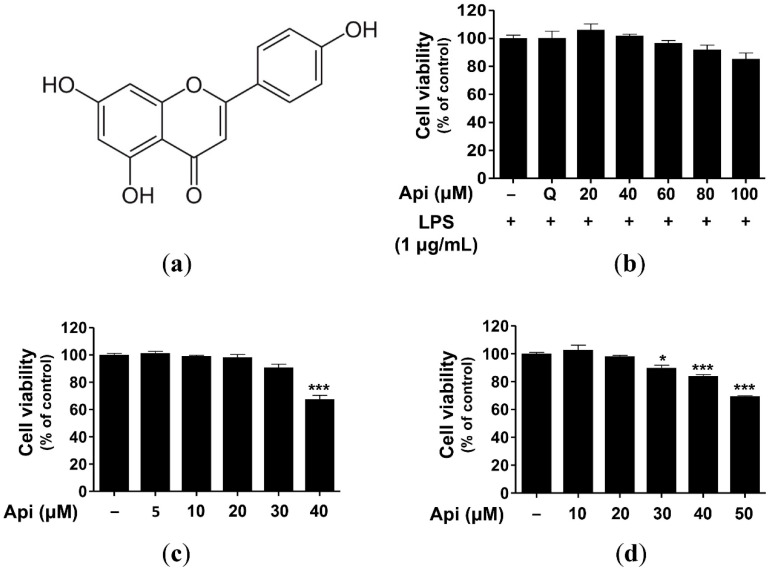
Molecular structure (**a**) and cytotoxic effects of apigenin on RAW264.7 (**b**), RBL-2H3 (**c**), and HaCaT cells (**d**). Cells were treated with various concentrations of apigenin (5, 10, 20, 30, 40, 50, 60, 80, and 100 μM) for 24 h. The data were analyzed using one-way analysis of variance (ANOVA) followed by Tukey’s test. * *p* < 0.05, *** *p* < 0.001 versus LPS-exposed cells without apigenin treatment. Api, apigenin; LPS, lipopolysaccharide; Q, quercetin (15 μM).

Flavonoids comprise sugar-linked glycosides and aglycone. Several flavonoids are found in nature that are formed by different combinations of aglycone and the attached sugar moiety [57]. Apigenin is a secondary plant metabolite with a molecular formula C_15_H_10_O_5_ (Figure 1a). Barley sprout is rich in the flavone family of apigenin, including apigenin 6-*C*-arabinoside-8-*C*-glucoside (isoschaftoside), apigenin 6-*C*-glucoside-8-*C*-arabinoside (schaftoside), apigenin 6-*C*-glucoside (isovitexin), isovitexin 7-*O*-(6″-O-feruloyl) glucoside (6″-*O*-feruloylsaponarin), isovitexin 7-*O*-(6″-O-feruloyl) glucoside-4′-*O*-glucoside, isovitexin 7-*O*-(6″-*O*-sinapoyl) glucoside (6″-*O*-sinapoylsaponarin), isovitexin 7-*O*-glucoside (saponarin), and isovitexin 7-*O*-rutinoside [52,53]. 

Several studies have reported the mutagenic effects of flavonoids that are associated with their pro-oxidant activities [58,59,60]. Thus, we evaluated the cytotoxicity of apigenin in RAW264.7, RBL-2H3, and HaCaT cells. As shown in Figure 1b–d, 40 and 30 μM apigenin induced significant cytotoxicity in RBL-2H3 (67.5%, *p* < 0.001) and HaCaT (89.9%, *p* < 0.05) cells, respectively. However, apigenin had no effect on RAW264.7 cells, even at a concentration of 100 μM. Therefore, in the subsequent experiments, RAW274.7, RBL-2H3, and HaCaT cells were treated with nontoxic concentrations of apigenin.

### 2.2. Effects of Apigenin on NO Production and β-Hexosaminidase Release

**Figure 2:**
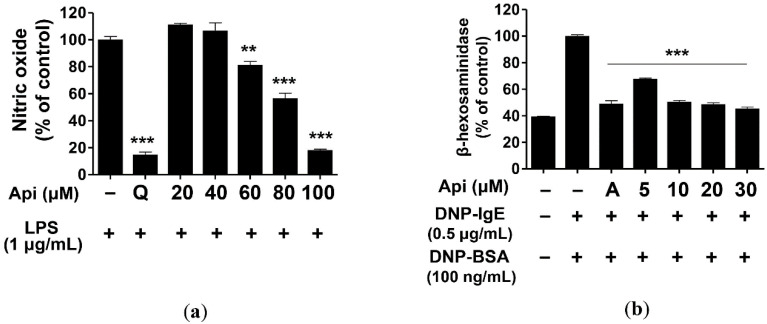
Effects of apigenin on nitric oxide production and β-hexosaminidase release of RAW264.7 (**a**) and RBL-2H3 cells (**b**), respectively. LPS- or IgE-induced cells were treated with various concentrations of apigenin (5, 10, 20, 30, 40, 60, 80, and 100 μM). Api, apigenin; A, cyclosporine A (1 μg/mL); LPS, lipopolysaccharide; Q, quercetin (15 μM). The data were analyzed using one-way analysis of variance (ANOVA) followed by Tukey’s test. ** *p* < 0.01, *** *p* < 0.001 versus LPS-stimulated cells without apigenin treatment.

Macrophages produce and secrete secondary mediators such as NO, PGE2, leukotriene, and proinflammatory cytokines. These substances play an important role in the regulation of innate and epigenetic immunity [23]. However, their overproduction may lead to bacterial sepsis, rheumatoid arthritis, chronic inflammation, and autoimmune diseases [61]. We evaluated the inhibitory activity of apigenin on NO production and found that apigenin significantly inhibited NO production in RAW264.7 cells as compared with LPS (positive control). Moreover, 100 μM apigenin could inhibit NO production in RAW264 cells at a level similar to that observed with quercetin (15 μM) [62], which exhibits excellent inhibitory activity on NO production (Figure 2a). 

β-Hexosaminidase is present in the granules of mast cells and basophils and is secreted in response to allergic reactions such as asthma and rhinitis. It serves as an indicator of degranulation and is useful for measuring the bioactivity of allergen inhibitors [63]. RBL-2H3 cells were treated with the indicated concentrations (5, 10, 20, and 30 μM) of apigenin, and the inhibition of degranulation was measured. Apigenin inhibited the degranulation of β-hexosaminidase in RBL-2H3 cells at a significant level at all concentrations, of which 10, 20, and 30 μM doses exerted inhibitory effects similar to those of cyclosporine A (1 μg/mL) [64] (Figure 2b). Thus, apigenin significantly inhibited NO production and β-hexosaminidase degranulation in LPS-induced RAW264.7 and IgE-induced RBL-2H3 cells, respectively.

### 2.3. Effects of Apigenin on Cytokines and MAPK Signaling Pathways in RAW264.7 Cells

**Figure 3:**
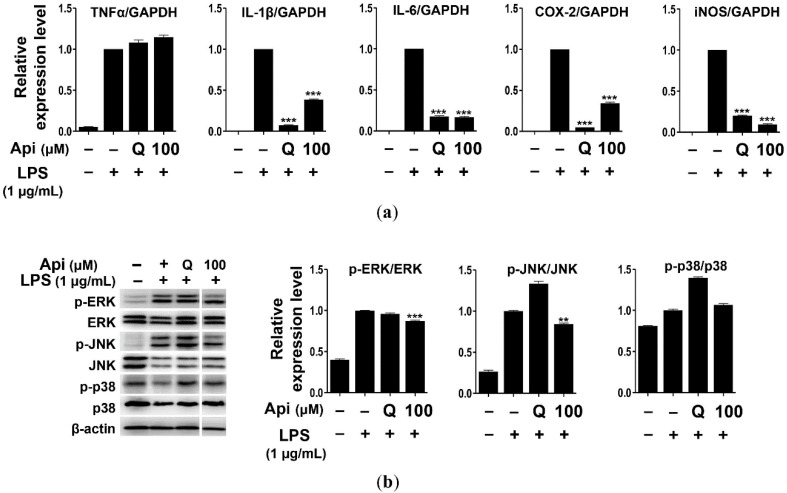
Effects of apigenin on the expression of cytokines (**a**) and mitogen-activated protein kinase (MAPK) signaling molecules (**b**) in RAW264.7 cells. Cells were treated with apigenin (100 μM) for 24 h. Api, apigenin; LPS, lipopolysaccharide; Q, quercetin (15 μM). The data were analyzed using one-way analysis of variance (ANOVA) followed by Tukey’s test. ** *p* < 0.01, *** *p* < 0.001 versus LPS-induced cells without apigenin treatment.

In general, inflammatory mediators such as NO and PGE2 are inevitably accompanied by inflammatory cytokines, including TNF-α, IL-1β, and IL-6 [28,29]. TNF-α is primarily produced by activated macrophages but may also be produced by the lymphoid, mast, and endothelial cells [23,28]. In particular, TNF-α expression is upregulated in response to LPS stimulation [65]. IL-1β is a representative preinflammatory cytokine that is very closely related to TNF-α. In general, IL-1β is necessary for cell growth or maintenance of homeostasis at low concentrations but can exacerbate human disease when overproduced during inflammatory reactions, wounds, or immunological stimuli [66]. iNOS is normally absent in cells, but its expression is induced by NF-κB. iNOS produces high levels of NO for an extended time period. This is an important mechanism for the overproduction of inflammatory mediators by LPS or cytokine in macrophages [67]. COX-1 acts on normal biological functions such as platelet formation, maintenance of mucosal integrity in the gastrointestinal tract, and kidney function. Many anti-inflammatory drugs act by inhibiting PG synthesis through the suppression of COX-2 production or activity [68]. Apigenin (100 μM) was found to significantly inhibit the expression of TNF-α, IL-1β, IL-6, iNOS, and COX-2 in LPS-induced RAW264.7 cells (Figure 2a). Further, the expression of IL-6 and iNOS decreased following treatment with apigenin, which was as effective as, or even more effective than, quercetin (5 μM). However, both apigenin and quercetin failed to decrease the expression of TNF-α (Figure 3a), consistent with a previous report that showed the absence of any effect of quercetin on the expression of TNF-α [69].

MAPK is a serine-threonine kinase that plays an important role in the regulation of cell growth and differentiation as well as cellular responses to cytokines and stress [21,70]. ERK is widely activated by stimulators and can phosphorylate various transcription factors. p38 and JNK are part of the stress response pathways and are activated by factors such as inflammatory cytokines [71]. The JNK signaling pathway is activated in cells in response to immune-inflammatory stimuli such as LPS and TNF-α, and is involved in cell morphology and cytokine transcription [72,73]. We investigated the effect of apigenin (100 μM) on the MAPK signaling pathway, and found that the phosphorylation of ERK and JNK was inhibited in LPS-induced RAW264.7 cells (Figure 3b). These results suggest that apigenin inhibits the expression of inflammatory mediators such as IL-1β, IL-6, iNOS, and COX-2 in RAW264.7 cells through the suppression of the MAPK signaling pathway via ERK and JNK phosphorylation, consequently exhibiting anti-inflammatory effects.

### 2.4. Effects of Apigenin on Cytokines, MAPK, and Allergic Signaling Pathways in RBL-2H3 Cells

**Figure 4:**
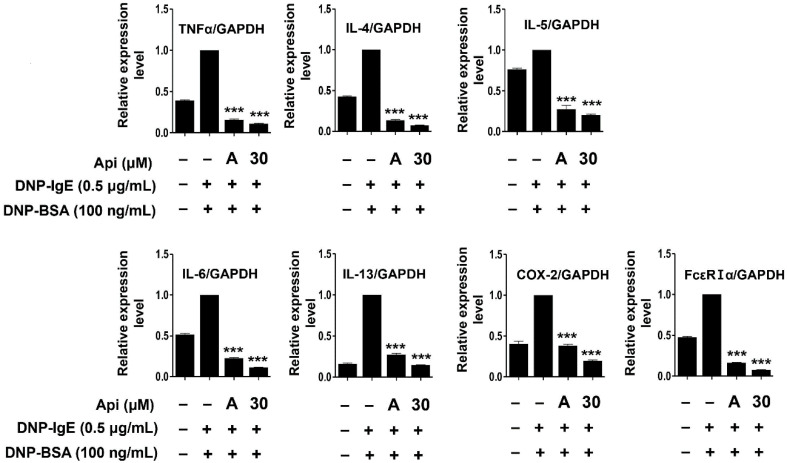
Effects of apigenin on the mRNA transcription of cytokines and FcεRIα in RBL-2H3 cells. Cells were treated with apigenin (30 μM), 0.5 μg/mL DNP-IgE, and 100 ng/mL DNP-BSA. Api, apigenin; A, cyclosporine A (1 μg/mL). The data were analyzed using one-way analysis of variance (ANOVA) followed by Tukey’s test. *** *p* < 0.001 versus DNP-IgE- and DNP-BSA-treated cells without exposure to apigenin.

Mast cells show surface expression of FcεRI, which is activated upon binding to the antigen-crosslinked IgE and mediates degranulation. Activation by crosslinking with IgE results in the production and secretion of lipid mediators that sustain the inflammatory response through subsequent reactions involving synthesis and secretion of various cytokines [74]. Activation of mast cells is known to increase the expression of the αβγ subunit of FcεRI [75]. Mast cell activation and inflammatory response are regulated by the production and secretion of cytokines, including IL-1β, IL-2, IL-3, IL-4, IL-5, IL-6, IL-10, IL-12, IL-13, TNF-α, and granulocyte-macrophage colony-stimulating factor (GM-CSF) [76]. TNF-α is a major cytokine secreted and stored by mast cells that increases the endothelial and epithelial cell adhesion molecules and enhances airway hypersensitivity. IL-6 is expressed during the acute phase of inflammation and plays a role in the development and exacerbation of Th2-mediated diseases such as allergic airway inflammation and asthma. IL-5 is an essential cytokine for the development and survival of eosinophils [77,78,79], while IL-4 promotes B cell differentiation, leading to IgE synthesis and immediate hypersensitivity reactions along with the induction of vascular cell adhesion molecule (VCAM)-1 expression in endothelial cells. It also induces the expression of IL-5, which is essential for the differentiation of Th2 cells and eosinophils [80]. IL-13 is a Th2 cytokine that binds to the α-chain of the IL-4 receptor by sharing a receptor with IL-4 and is an important regulator that induces IgE production along with IL-4 [81,82]. In general, the expression of IgE is upregulated in AD. The extrinsic types respond immediately to inhalable or food antigens, while the intrinsic types are not related to hypersensitivity to antigens when IgE level is in normal range [83,84,85]. In particular, the expression of IL-4 and IL-13 is upregulated in the extrinsic type as compared to that in the intrinsic type [86,87,88]. As shown in Figure 4, the expression of TNF-α, IL-4, IL-5, IL-6, IL-13, COX-2, and FcεRIα in RBL-2H3 cells activated by 2,4-dinitrophenylated albumin from bovine serum (DNP-BSA) and DNP-IgE was significantly inhibited by apigenin (30 μM). As the effect of apigenin was similar to that observed with cyclosporine A (1 μg/mL), apigenin would be potentially effective in mediating abnormal immune responses owing to excessive cytokine expression.

**Figure 5:**
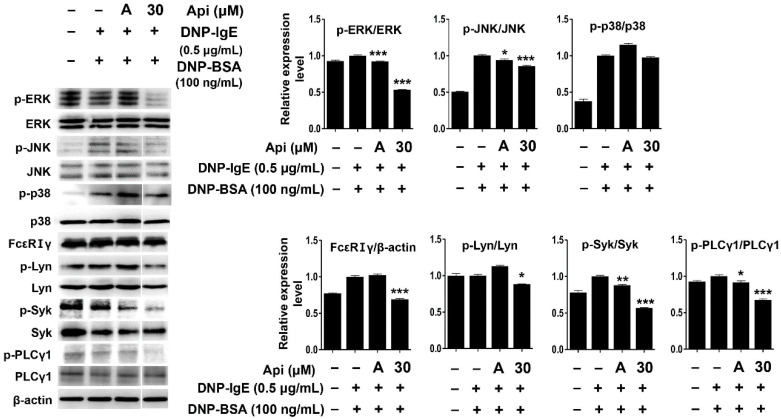
Effects of apigenin on mitogen-activated protein kinase (MAPK) and IgE-activated signaling molecules in RBL-2H3 cells. Cells were treated with apigenin (30 μM), 0.5 μg/mL DNP-IgE, and 100 ng/mL DNP-BSA. Api, apigenin; A, cyclosporine A (1 μg/mL). The data were analyzed using one-way analysis of variance (ANOVA) followed by Tukey’s test. * *p* < 0.05, ** *p* < 0.01, *** *p* < 0.001 versus DNP-IgE- and DNP-BSA-treated cells without apigenin exposure.

The mast cell signaling pathway is activated after the binding of the antigen-IgE complex to the α-subunit of FcεRI, resulting in the activation of Lyn (Src-family kinase) bound to the β-subunit of FcεRI and the subsequent phosphorylation and activation of the γ-subunit [15,16,17,89]. The β- and γ-subunits of FcεRI carry an immunoreceptor-based activation motif (ITAM) that is phosphorylated by Lyn kinase. Phosphorylation of tyrosine in the ITAM motif provides a binding site for Syk kinase and induces the activation of Syk kinase by structural modification. Activated Syk sequentially activates various sub-signaling molecules such as phospholipase Cγ1 (PLCγ1) and MAPKs to secrete mediators that induce various allergic responses [15,16,17,90,91]. Apigenin significantly reduced the expression of FcεRIα/γ protein and inhibited the phosphorylation of Lyn, Syk kinase, and PLCγ1, which are essential for the activation of RBL-2H3 cells (Figure 5). 

MAPKs play an important role in the production of various cytokines, including TNF-α and IL-4, upon antigenic stimulation of mast cells [19,20,21,92]. We performed immunoblot analysis to evaluate the effect of apigenin on the phosphorylation of MAPK signaling molecules. As shown in Figure 5, the phosphorylation of ERK and JNK, but not p38, significantly decreased after apigenin treatment. Thus, the apigenin-mediated inhibition of the phosphorylation of signaling molecules and the expression of cytokines in RBL-2H3 cells is suggestive of its application as an effective agent to control inflammation and allergic responses.

### 2.5. Effects of Apigenin on HaCaT Cells

**Figure 6:**
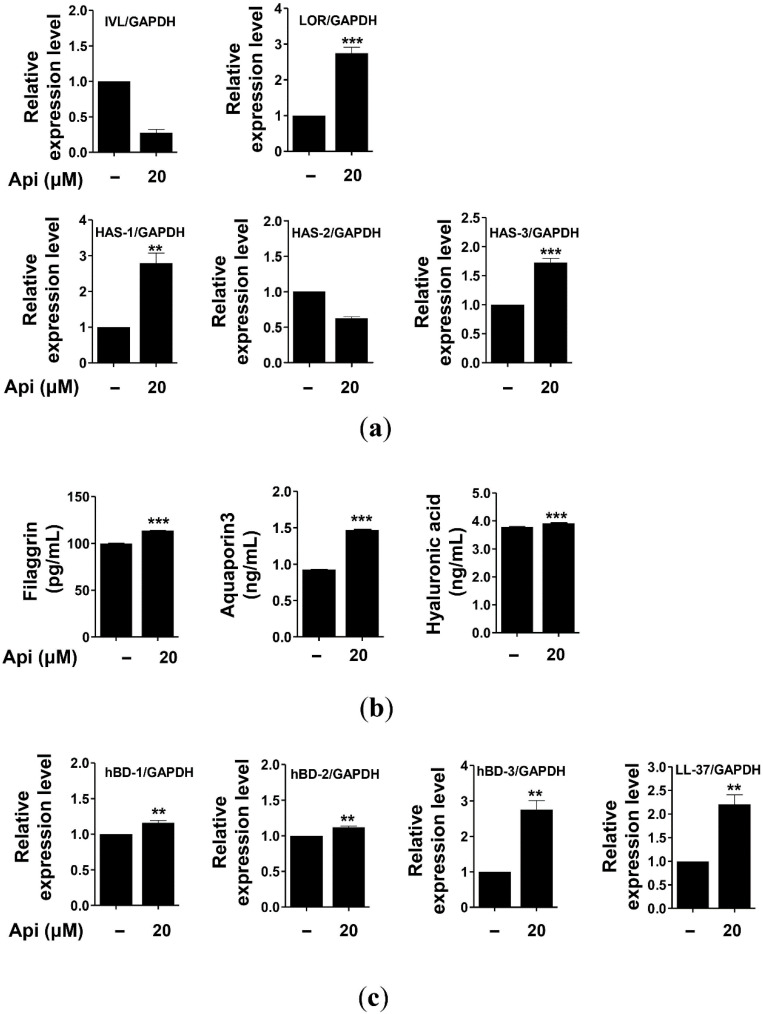
Effects of apigenin on the expression of the genes related to the physical barrier (**a**,**b**) and chemical barrier (**c**) functions of HaCaT cells. Cells were treated with apigenin (20 μM) for 24 h. Api, apigenin; IVL, involucrin; LOR, loricrin. The data were analyzed using *t*-test. ** *p* < 0.01, *** *p* < 0.001 versus cells without apigenin treatment.

Given the importance of the function of the skin barrier, studies have been directed to develop functional substances related to the skin based on the known antioxidant mechanisms. The relationship between AD and mutations of the filaggrin gene has been recently reported. Chronic skin diseases such as psoriasis are thought to be related to the decrease in the expression of antimicrobial peptides [93,94,95]. Damage to the skin barrier may facilitate the penetration of allergens and induce skin diseases such as AD. Filaggrin and involucrin are the major constituent proteins that are downregulated in response to the damage to the skin barrier. Mutations of filaggrin have been reported to be the main causes of AD, asthma, and allergic rhinitis [10,32,42]. AQP is a water- and glycerol-carrying protein produced by keratinocytes that is involved in the movement and differentiation of keratinocytes. It has been reported to play an important role in restoring skin barrier functions [34,35,36,37,38,39,96,97]. Any decrease in the biosynthesis of HA, a major component of the skin extracellular matrix that acts as a moisturizing barrier, may produce wrinkles and reduce skin elasticity, consequently leading to premature aging of the skin, psoriasis, and dermatitis. Therefore, maintenance of HA level is important to prevent skin aging and diseases [33]. HA is synthesized by HAS in keratinocytes; three *HAS* genes have been known so far, namely, *HAS-1*, *HAS-2*, and *HAS-3* [98,99]. The decrease in the expression of *HAS* genes is known to induce skin aging, including defects in the moisturizing barrier, atrophy of the epidermis, wrinkle formation, decrease in skin moisture, and reduced elasticity [33,99]. Some enzymes play a crucial role in HA synthesis, and research has been conducted to increase the synthesis of HA by promoting the expression of HAS [100,101,102]. We examined the effect of apigenin on the expression of the genes encoding involucrin, loricrin, filaggrin, HAS-1, HAS-2, HAS-3, and AQP3 in HaCaT cells using real-time quantitative polymerase chain reaction (PCR). As shown in Figure 6, apigenin significantly increased the expression of the genes encoding loricrin, HAS-1, and HAS-2, but not involucrin and HAS-2, in HaCaT cells. We also evaluated the effects of apigenin on the biosynthesis of filaggrin, AQP3, and HAS by enzyme-linked immunosorbent assays (ELISAs), and found these three protein levels to be significantly increased. Therefore, apigenin could improve and strengthen the physical barrier function of the skin by promoting the expression of the constituents in HaCaT cells. 

The skin acts as a chemical barrier against the invading pathogens by producing antimicrobial peptides such as HBD and cathelicidin (LL-37), which exert antimicrobial activities in the keratinocytes of the epidermis [40,41,42,43]. HBD is a cationic peptide of 3-4 kDa; 11 types of HBD have been reported in humans [1]. The ones that are mainly expressed in the skin are HBD-1, HBD-2, and HBD-3 [93,103,104]. HBD-1 is constitutively expressed under normal conditions in the epithelium and sweat glands, but the expression of HBD-2 and HBD-3 is observed in the skin or epithelial cells following bacterial infection, cytokine stimulation (IL-1β and TNF-α), and keratinocyte differentiation [105]. LL-37 is present in the lamellar body of keratinocytes along with HBD-2 and exerts strong antimicrobial activity against bacteria, fungi, and viruses. Further, LL-37 is also involved in maintaining the normal function of the skin barrier wall [106]. These antimicrobial peptides are important components of the innate immunity that are rapidly expressed after a bacterial or viral infection, as well as under normal conditions to protect from external infections [107]. Therefore, the decrease in the expression of antimicrobial peptides reduces the innate immunity and weakens resistance to pathogens, leading to psoriasis, acne, and chronic inflammatory skin diseases [93,95]. We evaluated the effect of apigenin on the expression of antimicrobial peptides, including HBD-1, HBD-2, HBD-3, and LL-37, of HaCaT cells using real-time quantitative PCR. As shown in Figure 6, the expression of the genes encoding these four proteins was significantly upregulated after treatment with apigenin. Thus, apigenin can be used as an agent to improve the physical and chemical barrier functions of the skin and relieve chronic inflammatory skin diseases, including psoriasis, acne, and AD.

## 3. Materials and Methods 

### 3.1. Reagents

Dulbecco’s modified Eagle’s medium (DMEM), antibiotics (penicillin and streptomycin), and trypsin-ethylenediaminetetraacetic acid (EDTA) were purchased from Gibco BRL (Grand Island, NY, USA). Fetal bovine serum (FBS) was obtained from Biowest (Kansas City, MO, USA), and 3-(4,5-dimethylthiazol-2-yl)-2,5-diphenyltetrazolium bromide (MTT), LPS, 4-nitrophenyl n-acetyl-b-d-glucosaminide (p-NAG), and monoclonal anti-DNP-IgE were supplied by Sigma–Aldrich (St. Louis, MO, USA). DNP-BSA was procured from Invitrogen (Gaithersburg, MD, USA). Primary antibodies against p-p38, p38, p-JNK, JNK, p-ERK, ERK, p-Lyn, Lyn, p-Syk, Syk, p-PLCγ, PLCγ, and β-actin were obtained from Cell Signaling Technology (Danvers, MA, USA), and FcεRIγ, from LSBio (Seattle, WA, USA). SensiFAST SYBR No-ROX kit mix was purchased from Biolines (Seoul, Korea), and human filaggrin, AQP3, and HA ELISA kits were obtained from CUSABIO (Seoul, Korea). Rat basophilic leukemia (RBL-2H3, ATCC^®^ CRL-2256) and murine macrophage (RAW264.7, ATCC^®^ TIB-71) cells were procured from the American Type Culture Collection (ATCC), while human immortalized keratinocyte (HaCaT) cells were obtained from Prof. Lee of Chosun University in Korea.

### 3.2. Cell Culture

RBL-2H3, RAW264.7, and HaCaT cells were maintained in DMEM containing 10% FBS and 1% antibiotics (penicillin and streptomycin). The cells were incubated at 37 °C in a 5% CO_2_ humidified incubator.

### 3.3. Cell Viability Assay

RAW264.7, RBL-2H3, and HaCaT cell viability were analyzed using the MTT assay [108]. RAW264.7 cells were treated with quercetin (15 μM) and apigenin (20, 40, 60, 80, and 100 μM) in the absence or presence of LPS (1 μg/mL). RBL-2H3 and HaCaT cells were treated with various concentrations of apigenin (RBL-2H3: 5, 10, 20, 30, and 40 μM; HaCaT: 10, 20, 30, 40, and 50 μM). After incubation for 24 h, 0.5 mg/mL of MTT solution was added to each well. The supernatants were discarded, and the resulting formazan crystals were dissolved in dimethyl sulfoxide (DMSO) and transferred to a 96-well plate. Absorbance (570 nm) was measured using a microplate reader (TECAN, Männedorf, Switzerland).

### 3.4. NO and β-Hexosaminidase Release Assay

RAW264.7 cells were seeded in 24-well plates in 5 × 10^4^ cells/well and incubated for 24 h. The cells were treated with LPS (1 μg/mL) and apigenin (20, 40, 60, 80, and 100 μM) for 24 h. The supernatant was mixed with Griess reagent in a 96-well plate for 10 min, and the absorbance was measured at 530 nm using microplate reader. The amount of NO produced was calculated using a sodium nitrite (NaNO_2_) standard curve. 

For β-hexosaminidase release assay, RBL-2H3 cells were seeded in 24-well plates at 2 × 10^5^ cells/well and treated with 0.5 μg/mL DNP-IgE for 24 h. The medium was removed and washed with Tyrode buffer (119 mM sodium chloride (NaCl), 5 mM potassium chloride (KCl), 2.5 mM calcium chloride (CaCl_2_), 1.19 mM magnesium sulfate (MgSO_4_), 10 mM HEPES, 5 mM glucose, and 1 mg/mL BSA, pH 7.3). The cells were incubated with apigenin (5, 10, 20, and 30 μM) for 20 min, and then with 100 ng/mL DNP-BSA for 1 h. The supernatants (50 μL) were incubated with a substrate buffer (3.3 mM p-nitrophenyl-N-acetyl-β-D-glucosaminide, pH 4.5) in 96-well plates at 37 °C for 1 h. The reaction was terminated using 100 μL a stop solution (0.1 M sodium carbonate (Na_2_CO_3_)/sodium bicarbonate (NaHCO_3_), pH 10.2), and the absorbance was measured at 407 nm using a microplate reader. β-Hexosaminidase release activity was measured as per the following equation: β-hexosaminidase release activity (%) = (OD_407_ of sample/OD_407_ of control) × 100. 

### 3.5. Real-Time Quantitative PCR

Total RNA was prepared from cells using Trizol Reagent (Thermo Scientific, Seoul, Korea) according to the manufacturer’s instructions. Total RNA (1 μg/mL) and 50 μM oligo-dT primer were mixed in 15 μL of DEPC-water and reacted at 70 °C for 5 min. After the reaction, 2 μL of 100 mM dithiothreitol, 2 μL of 10 mM dNTP, 5 μL of 5× RT buffer, and 1 μL of 200 unit/μL M-MLV RTase (Bioneer, Daejeon, Korea) were added to synthesize cDNA in a reaction at 25 °C for 5 min, followed by 42 °C for 60 min and 70 °C for 15 min. Real-time quantitative PCR was performed on Rotor-Gene 6000 (Qiagen, Seoul, Korea) using SensiFast SYBR No-ROX kit, 10 pM of each primer (Supplementary Table S1), and 100 ng of cDNA. After amplification, the melting curve analysis was performed to confirm the specificity of the reaction. The end-point cycle threshold (Ct) used for real-time PCR quantification was defined as the number of PCR threshold cycles. The relative quantification of the target gene expression level was assessed using the ^ΔΔ^C*t* method.

### 3.6. Western Blot Analysis

Cell pellets were resuspended in 50 μL radioimmunoprecipitation (RIPA) lysis buffer (Thermo Scientific, Seoul, Korea) containing 1× protease inhibitor cocktail and phosphatase inhibitor cocktail (GenDEPOT, Seoul, Korea). Total protein concentration was determined using the Bradford 1× dye reagent (Bio-Rad, Seoul, Korea) at 595 nm. Equal amounts of protein (8 μg) were electrophoresed using sodium dodecyl sulfate-polyacrylamide gel electrophoresis (SDS-PAGE), and the separated protein bands were transferred onto a polyvinylidene fluoride (PVDF) membrane (Merck Millipore, Seoul, Korea). The membrane was blocked with 5% BSA (GenDEPOT, Seoul, Korea) and incubated with 1:1000 diluted primary antibodies (p-p38, p38, p-JNK, JNK, p-ERK, ERK, p-Lyn, Lyn, p-Syk, Syk, p-PLCγ, PLCγ, FcεRIγ, and β-actin) at 4 °C for 16 h. Western blot signals were visualized using horseradish peroxidase (HRP)-conjugated secondary antibodies (Santa Cruz Biotech, Dallas, TX, USA) and developed with EZ-western Lumi Femto^TM^ Kit (Dogen, Seoul, Korea). The samples were scanned using the C-Digit blot scanner (LI-COR Biosciences, Lincoln, NE, USA), and quantified using the ImageJ software (National Institutes of Health, Bethesda, MD, USA).

### 3.7. ELISA

FLG, AQP3, and HA production was measured using an ELISA kit as per the manufacturer’s instructions (CUSABIO, Seoul, Korea). Briefly, HaCaT cells (6 × 10^4^ cells/well) were seeded and treated with apigenin (20 μM) for 24 h. FLG, AQP3, and HA levels were measured at 450 nm wavelength using a microplate reader (TECAN, Männedorf, Switzerland).

### 3.8. Statistical Analysis

All data are expressed as mean ± standard deviation (SD) of three independent experiments. Statistical significance from the control group was evaluated using one-way analysis of variance (ANOVA), followed by Tukey’s test and *t*-test using Prism software (GraphPad Software Inc., La Jolla, CA, USA). 

## 4. Conclusions

In this study, we evaluated the effect of apigenin on major mediators of inflammatory and allergic responses in RAW264.7 and RBL-2H3 cells. Apigenin (100 μM) effectively inhibited NO production and cytokine expression (IL-1β, IL6, COX-2, and iNOS) as well as the phosphorylation of ERK and JNK associated with MAPK signaling pathway in RAW264.7 cells. Apigenin (30 μM) also inhibited the expression of cytokines (TNF-α, IL-4, IL-5, IL-6, IL-13, and COX-2) and FcεRIα/γ as well as the phosphorylation of signaling molecules (Lyn, Syk, PLCγ1, ERK, and JNK) corresponding to allergic responses pathway in RBL-2H3 cells. Moreover, apigenin (20 μM) significantly induced gene or protein expression (filaggrin, loricrin, AQP3, HA, HAS-1, HAS-2, and HAS-3) in HaCaT cells of molecules that play an important role in the physical barrier and water retention properties of the skin. Further, it increased the expression of antimicrobial peptides (HBD-1, HBD-2, HBD-3, and LL-37) that play an important role in acting as chemical barriers of HaCaT cells. However, apigenin has a very low solubility in water. Therefore, it is necessary to develop delivery systems such as liposomes, polymeric micelles, nanosuspension in order to improve absorption and bioavailability in consideration of absorption, distribution, metabolism, and excretion (ADME). Although additional research is warranted, the results of the present study highlight apigenin as a potential candidate for alleviating immune-related diseases and AD.

## Supplementary Materials

Supplementary materials can be found at https://www.mdpi.com/1422-0067/21/13/4620/s1. Table S1: Primers used in this study.
